# Inflammatory brain lesions preceding primary central nervous system lymphoma: a case report and genetic analysis

**DOI:** 10.1007/s10072-022-06587-7

**Published:** 2023-01-05

**Authors:** Zheng XiaoHong, Yin Shuo, Dong GeHong, Yang AnChao, Wang Ce, Duan YunYun, Wang Can, Huang SiJie, Chen Feng, Li WenBin

**Affiliations:** 1 Department of Neuro-Oncology, Cancer Center, Beijing Tiantan Hospital, Capital Medical University, Beijing, China; 2 Department of Pathology, Beijing Tiantan Hospital, Capital Medical University, Beijing, China; 3 Department of Neurosurgery, Beijing Tiantan Hospital, Capital Medical University, Beijing, China; 4 Department of Radiology, Beijing Tiantan Hospital, Capital Medical University, Beijing, China

**Keywords:** Primary central nervous system lymphoma, Inflammatory brain lesions, Sentinel lesions, Case report, Genetic testing

## Abstract

**Background:**

Primary central nervous system lymphoma (PCNSL) is an aggressive extranodal lymphoma exclusively occurring within the central nervous system. Inflammatory brain lesions as “sentinel lesions” of PCNSL are very rare. We present a rare case of PCNSL with preceding inflammatory lesions in an immunocompetent patient who underwent two biopsies, one craniotomy and two genetic testing.

**Case report:**

A 66-year-old male patient presented with left limb weakness and ataxia. Brain magnetic resonance imaging showed a contrast-enhancing lesion with perifocal brain edema in the near midline of right frontal lobe. Histological examination of a brain biopsy specimen revealed inflammatory lesion characteristics with infiltration of T-cell dominant lymphocytes and few B-cell. Given that the patient developed cerebral hematoma after biopsy, lesion resection by craniotomy was performed. An excised sample demonstrated mixed T-cell and B-cell infiltrating inflammatory lesions. Four months after total resection of the right frontal lobe lesion, another lesion appeared in the left frontal parietal lobe, which was diagnosed as diffuse large B-cell lymphoma by biopsy. In addition, genetic testing of the lesions at two different locations was performed, and the results showed that the inflammatory lesions had the same three gene (RELN, PCLO, and CREBBP) mutations as PCNSL. Interestingly, the three mutated genes are associated with tumor.

**Conclusion:**

Our present case is the first to demonstrate inflammatory brain lesions heralding PCNSL from genetic and pathological perspectives. This may help clinicians to select new auxiliary diagnostic methods for timely diagnosis of patients with suspected PCNSL.

## Introduction

Primary central nervous system lymphoma (PCNSL) is a rare and aggressive extranodal non-Hodgkin lymphoma (NHL) limited to the brain, spinal cord, leptomeninges, or eyes, accounting for approximately 2% of all primary central nervous system tumors [1] and 4–6% of extranodal lymphomas [2]. Unlike other brain tumors, patients with PCNSL had no survival benefit after gross total or subtotal resection [3]. Chemotherapy based on high-dose methotrexate (HD-MTX) is the first-line treatment for patients with newly diagnosed PCNSL, which improved the prognosis of patients with PCNSL. Thus, an early and accurate diagnosis of PCNSL is critical for treatment and prognosis.

Several case studies have demonstrated the brain “sentinel lesions” preceding the development of PCNSL [4–9]. In previous reports, the “sentinel lesions” are described as demyelinating, and T-cell dominant inflammatory lesions and PCNSL can develop in chronological order in different locations [4, 6, 8, 9]. It is unclear if the preceding inflammatory lesions are related to the development of PCNSL. Elucidating this relationship may be helpful to timely diagnosis and treatment of patients with PCNSL, thereby improving prognosis.

PCNSL is known to disappear temporarily after steroid therapy. Therefore, it is difficult to distinguish the “sentinel lesions” of PCNSL from demyelinating diseases following steroid treatment [6]. Currently, the development of next-generation sequencing (NGS) technology further increases the diagnostic accuracy of PCNSL [10]. In the present study, we report a rare case of PCNSL with preceding inflammatory lesions in an immunocompetent patient who underwent two biopsies, one craniotomy and two genetic analyses. Based on this exemplary case, we also discuss the phenomenon of PCNSL “sentinel lesions” from genetic and pathological perspectives.

## Case report

**Fig. 1:**
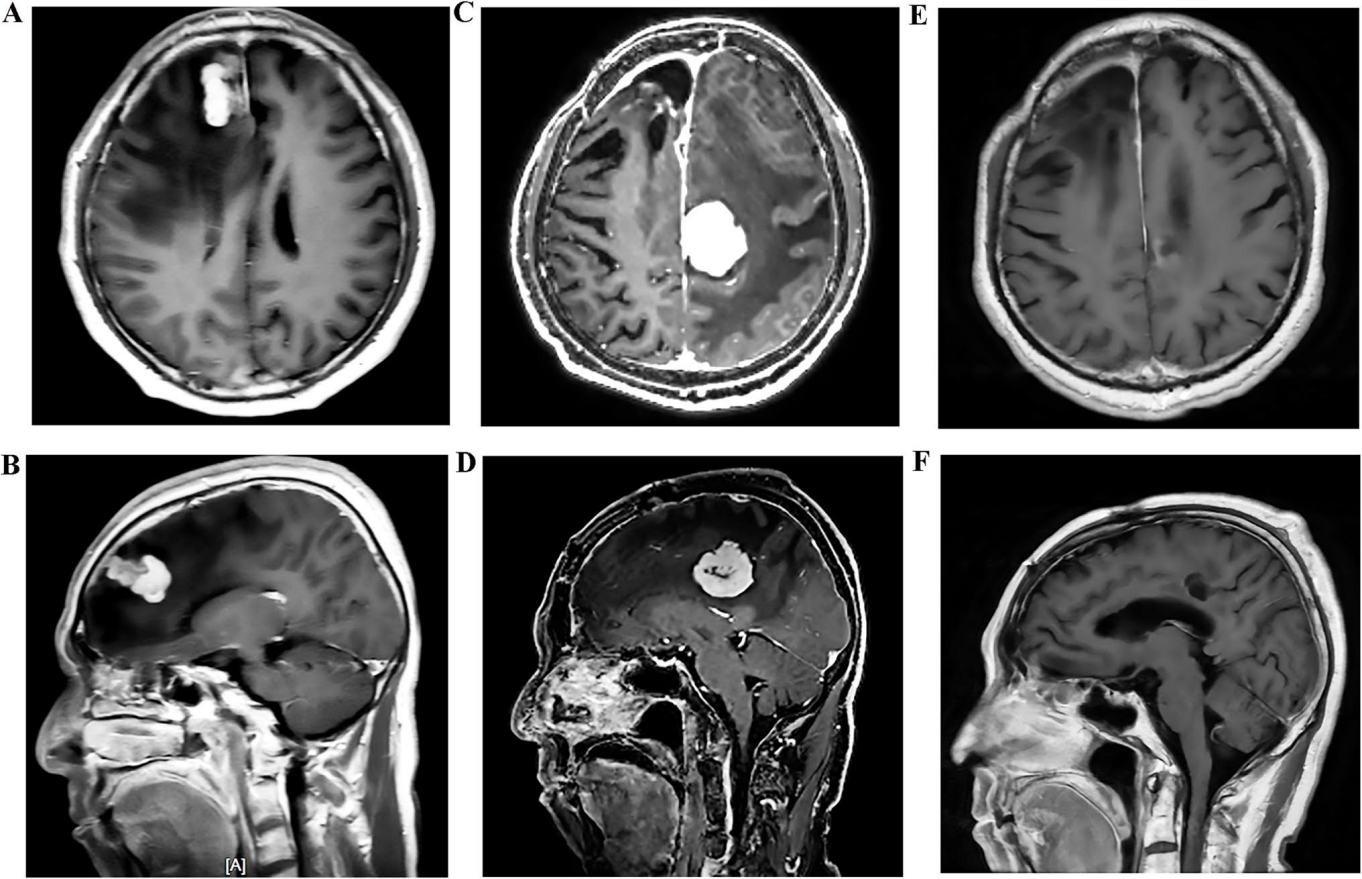
Brain MRI. T1-weighted MRI with gadolinium (**A**, **B**) showing an enhancing lesion in the near midline of right frontal lobe with perilesional edema. The pathology of this lesion suggested inflammation lesion. T1-weighted MRI with gadolinium (**C**, **D**) demonstrating the new enhancing lesion in the left frontal parietal lobe and was diagnosed as PCNSL. Follow-up T1-weighted MRI with gadolinium (**E**, **F**) revealing nearly complete resolution of the new lesion after chemotherapy therapy

**Fig. 2:**
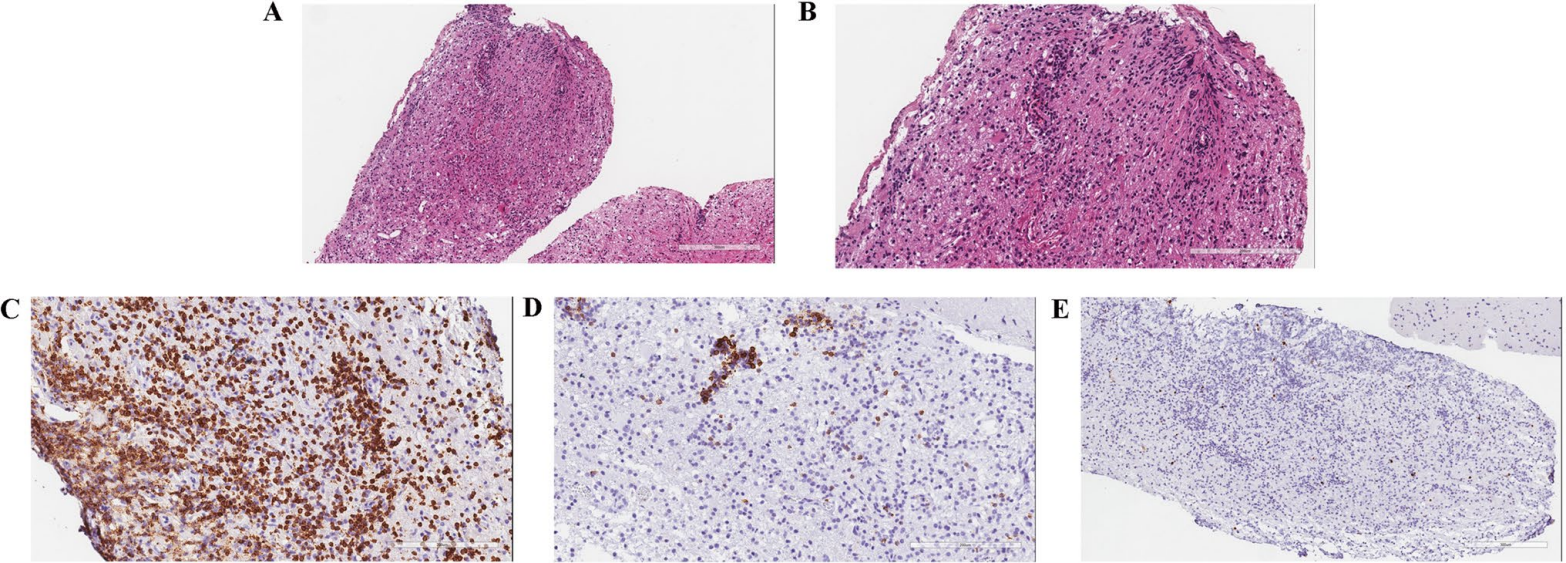
Histopathological and immunohistochemical findings in the first biopsy. Histopathological examination of the specimen (**A**, **B**) showing macrophages and lymphocytes infiltration in the perivascular space and glial stroma (hematoxylin and eosin staining, 100 × , 200 ×). Immunohistochemical staining showing mainly consisted of CD3 + lymphocytes (**C**, 200 ×) with few CD20 + (**D**, 200 ×) lymphocytes and low Ki-67 index (**E**, 100 ×). Scale bar: (**A**, **E**) 300 μm and (**B**, **C**, **D**, and **E**) 200 μm

An immunocompetent 66-year-old man initially presented in December 2020 with left limb weakness and ataxia. His past medical history included thyroidectomy and gastric ulcer perforation surgery. In February 2021, brain magnetic resonance imaging (MRI) showed a contrast-enhancing lesion with perifocal brain edema in the near midline of the right frontal lobe (Fig. 1A–B). Whole-body positron emission tomography/computed tomography (PET/CT) demonstrated increased fluorodeoxyglucose (FDG) metabolism in the right frontal lobe lesion. According to the imaging characteristics of brain MRI and PET/CT, the patient was suspected of PCNSL. Then, stereotactic biopsy was performed to further clarify the diagnosis. Corticosteroid treatment was not applied before the initial biopsy. Histopathological analysis revealed inflammatory changes in the brain tissue, accompanied by partial myelin destruction. The inflammatory infiltrate mainly consisted of CD3-positive T-lymphocytes and CD68-positive macrophages with only a few CD20-positive B-lymphocytes present and a low Ki-67 index (Fig. 2). Meanwhile, 93 tumor-associated genes were tested by NGS technology (Beijing GeneX Health Co., Ltd.). The result showed mutations in RELN, PCLO, and CREBBP genes, which were insufficient to diagnose PCNSL (Table 1).

**Table 1:** The genetic test results of the right frontal lobe lesion

Gene	Exon/intron	Mutation	Mutations in abundance
RELN	Exon30	p.V1470I	50.40%
PCLO	Exon7	p.A4279V	49.50%
PCLO	Exon2	p.Q375delinsPLGPAKPPAQH	32.30%
CREBBP	Exon2	p.A254T	47.30%

**Fig. 3:**
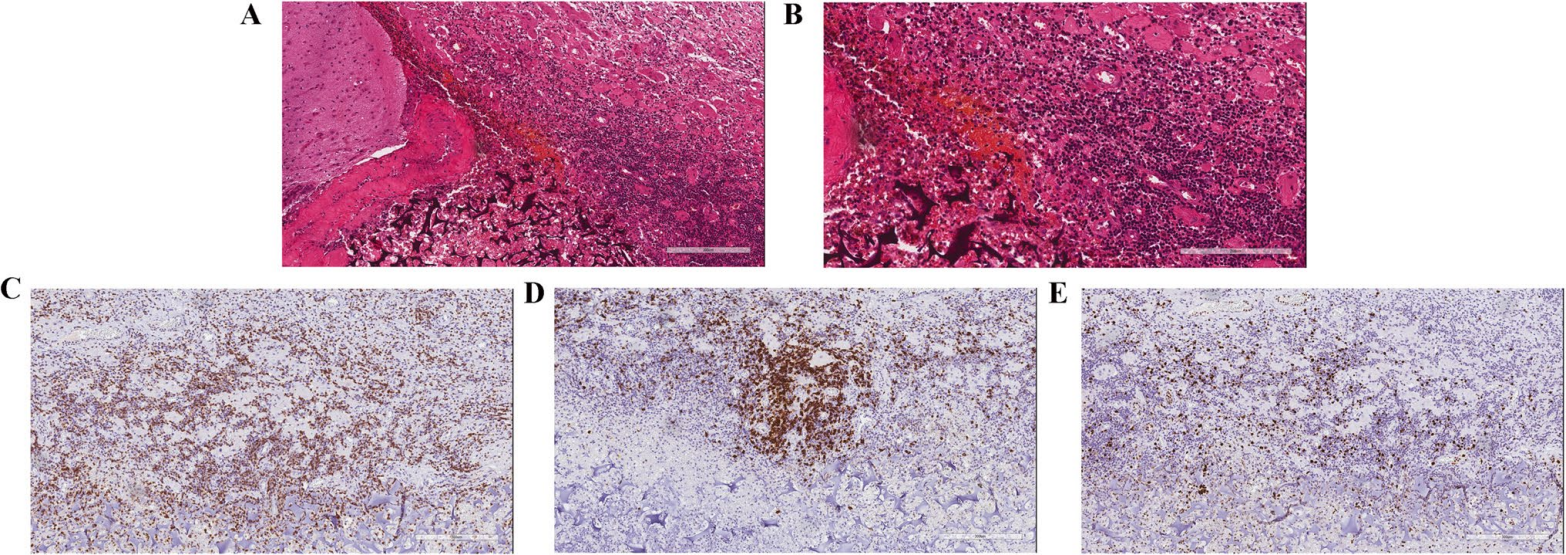
Histopathological and immunohistochemical findings of the tumor specimen obtained from craniotomy. Histopathological examination of the excised specimen (**A**, **B**) showing local area abscess formation with reactive gliosis and infiltration by macrophages, neutrophil, and lymphocytes (hematoxylin and eosin staining, 100 × , 200 ×). Immunohistochemical staining showing mixed CD3 + (**C**, 100 ×) and CD20 + (**D**, 100 ×) lymphocytic population with a high Ki-67 index (**E**, 100 ×). Scale bar: (**A**, **C**, **D**, **E**) 300 μm and (**B**) 200 μm

The patient developed cerebral hematoma after biopsy, and was treated with high-dose steroids (500 mg methylprednisolone daily for 5 days) and mannitol to reduce cranial pressure. Subsequently, steroid dosage was gradually reduced. In March 2021, brain CT showed no obvious absorption of the hematoma, and cerebrospinal fluid (CSF) examination suggested a bacterial infection. The biochemical results of CSF are as follows: glucose: 2.02 mmol/L, range 2.5–4.5 mmol/L; protein: 182.46 mg/dL, range 15–45 mg/dL; chloride: 124 mmol/L, range 118–132 mmol/L; and lactate: 7.9 mmol/L, range 1.1–2.4 mmol/L. The cytological results of CSF are as follows: appearance of CSF: yellowish turbidity; Pandy test: + ; Totle cell: 1843/μl; WBC (white blood cell): 1543/μl; PMN (polymorphonuclear leucocyte): 87.9%; and MN (monocyte): 12.1%. *S. aureus* was detected by CSF culture. To relieve symptoms of cerebral edema and hematoma, lesion resection by craniotomy and hematoma removal were performed after anti-infective treatment. Excision of the right frontal lobe lesion revealed local area abscess formation, and extensive lymphocytic infiltration composed of mixed CD3 + T-cells and CD20 + B-cells with high Ki-67 index (Fig. 3). The descriptive diagnosis was made as an inflammatory lesion with dense lymphocyte, plasma cell, and neutrophil infiltration. Due to insufficient diagnostic evidence for PCNSL and total resection of lesions, the patient did not receive anti-tumor therapy.

**Fig. 4:**
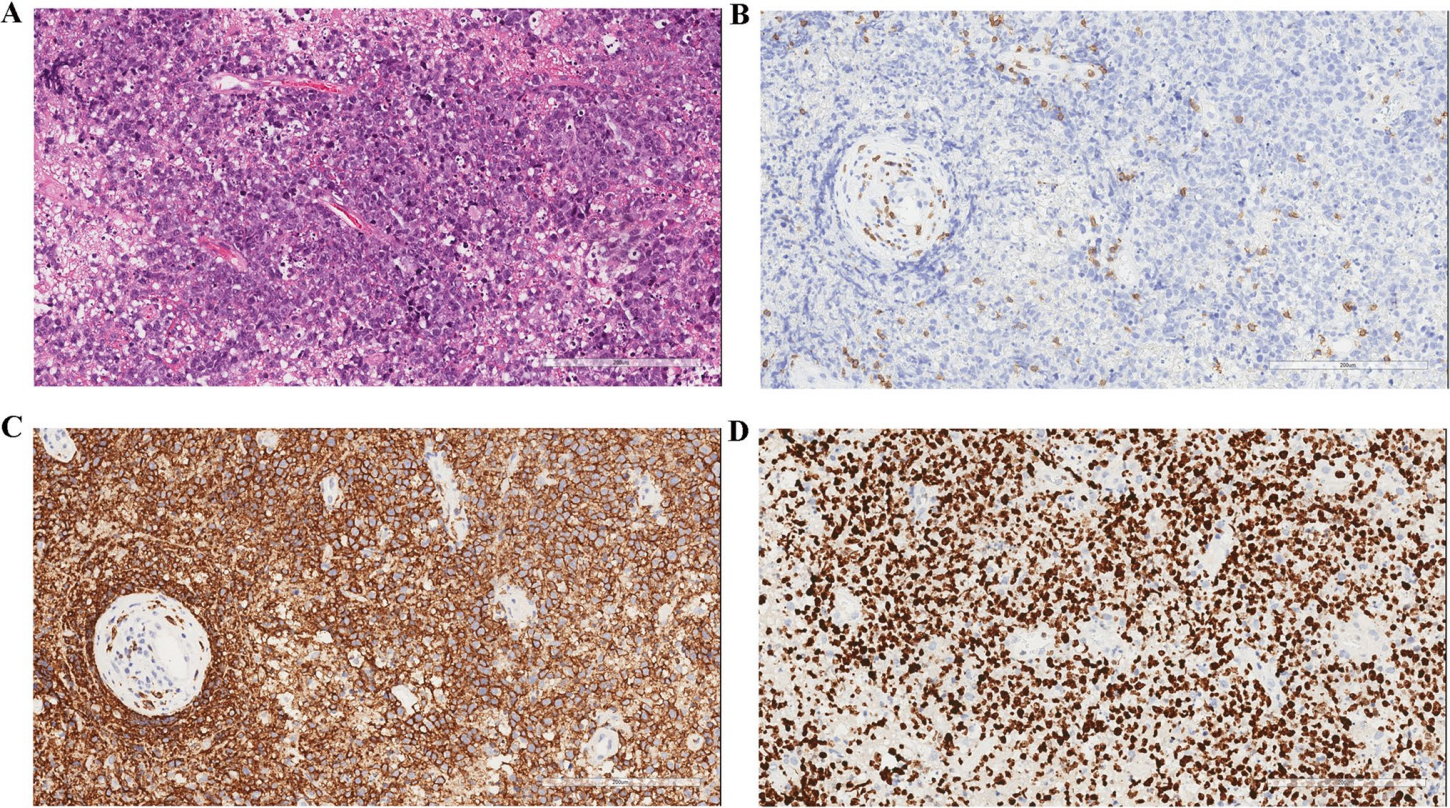
Pathological images of PCNSL. Histopathological examination of the second biopsy specimen (**A**) showing a densely cellular lesion composed of lymphoma cells with enlarged nuclei (hematoxylin and eosin staining, 200 ×). The majority of tumor cells were negative for CD3 (**B**, 200 ×) but positive for CD20 (**C**, 200 ×) with a high Ki-67 index (**D**, 200 ×). Scale bar: (**A**, **B**, **C**, **D**, **E**) 200 μm

In July 2021, 7 months after the initial attack, the patient suddenly developed right limb weakness and ataxia. Cranial MRI disclosed the right frontal lobular surgical remnant and a homogeneous enhanced new lesion in the left frontal parietal lobe with perifocal brain edema (Fig. 1C–D). With the suspect of PCNSL, stereotactic biopsy was performed again in August 2021. Biopsy of the new lesion revealed diffuse large B-cell lymphoma (Fig. 4). A subsequent systemic evaluation provided no evidence of a systemic lymphoma. Meanwhile, the biopsies were tested for 93 lymphoma-related genes using NGS technology. The result showed 23 gene mutations, including MYD88, CD79B, CDKN2A, PIM1, RELN, PCLO, CREBBP, and so on, supporting the diagnosis of diffuse large B-cell lymphoma (Table 2). Therefore, the condition was diagnosed as diffuse large B-cell type PCNSL. The patient was treated with intravenous high-dose methotrexate and rituximab for 6 cycles. This treatment provided clinical improvement and nearly complete remission of the lesions on subsequent brain MRI (Fig. 1E–F).

**Table 2:** The genetic test results of the left frontal parietal lobe lesion

Gene	Exon/intron	Mutation	Mutations in abundance
RELN	Exon30	p.V1470I	48.20%
PCLO	Exon7	p.A4279V	49.00%
CREBBP	Exon2	p.A254T	49.30%
CREBBP	Exon20	p.C1240Y	41.80%
CD79B	Intron	c.67 + 2 T > C	46.20%
CD79B	Exon5	p.Y196N	43.50%
CD79B	Exon4	p.G160V	42.70%
CD79B	Exon4	p.M163I	42.00%
MYD88	Exon5	p.L265P	89.10%
CDKN2A	Exon2	p.R80X	91.20%
ETV6	Exon6	p.G375R	62.70%
FOXO1	Exon1	p.M1T	46.20%
GNA13	Exon4	p.L256F	44.90%
GNA13	Exon4	p.F252C	44.20%
GNA13	Exon2	p.R103X	42.10%
GNA13	Exon1	p.C14Y	41.70%
GNA13	Exon1	p.E82Gfs*19	1.10%
PIM1	Exon4	p.G83D	46.00%
PIM1	Exon4	p.I175V	43.70%
PIM1	Exon2	p.E30Q	43.50%
PIM1	Exon4	p.S146R	41.70%
PIM1	Exon4	p.V151I	38.10%
SMARCA4	Exon29	p.E1360[5 > 4]	39.00%

## Discussion

This study presents a rare case of a patient who has PCNSL and with a preceding inflammatory brain lesion. Both two histological examinations of the same lesion (biopsy lesion, and total lesion after craniotomy) in the right frontal lobe showed the inflammatory brain lesion. Meanwhile, the genetic test results of the biopsy lesion did not support the diagnosis of PCNSL. However, 4 months after total resection of the right frontal lobe lesion, the new lesion appeared in the left frontal parietal lobe and was diagnosed as PCNSL. This rare case implies that the tumefactive inflammatory brain lesions are not only demyelinating lesions but also could be the “sentinel lesions” of PCNSL development. Therefore, close clinical observation is mandatory for patients who have such symptom.

The clinical course and imaging features of PCNSL may be difficult to distinguish from a space-occupying inflammatory lesion, such as tumor-like inflammatory demyelination. Thus, histological confirmation is required for the final diagnosis. Unlike other brain tumors, surgical resection is not beneficial for survival for PCNSL patients [3]. Almost all PCNSL cases that are surgically accessible in the brain are advised by stereotactic biopsies. In the present case, brain MRI and PET/CT suggested brain lesions like PCNSL, and stereotactic biopsy was performed to further clarify the diagnosis. Histopathological analysis revealed inflammatory changes in the brain tissue, accompanied by partial myelin destruction. Although the result of histological examination did not support the diagnosis of PCNSL, the possibility of PCNSL cannot be ruled out because the accurate histopathological diagnosis of stereotactic biopsy specimens cannot always be achieved. What’s more, most previous studies have reported that PCNSL can be associated with preceding inflammatory lesions [4–6, 9, 11, 12]. Thus, 93 tumor-associated genes were tested by NGS technology could be used to increase the diagnostic accuracy of the lesion. Unfortunately, the result was still insufficient to diagnose PCNSL.

Interestingly, three mutated genes (RELN, PCLO, and CREBBP) revealed by genetic testing have been reported to be associated with the development of tumors [13–17]. RELN is a known extracellular matrix protein expressed primarily in brain development, and it is believed to influence cell motility in the neural system [18, 19]. Okamura et al. reported the rate of hepatocellular carcinoma recurrence was associated with reduced RELN expression [14]. Piccolo is well studied in neuroscience as a large, multi-domain presynaptic cytomatrix protein. PCLO, as one of the most often altered or amplified genes in esophageal squamous cell carcinoma, encodes the Piccolo protein [15]. One study further reported that 17 of 49 patients with diffuse large B-cell lymphoma had nonsynonymous mutations in the PCLO gene [20]. These findings showed that PCLO may play critical roles in the initiation and progression of cancers. CREBBP belongs to the KAT3 family of histone/protein lysine acetyltransferases, and it encodes a highly conserved and ubiquitously expressed nuclear phosphoprotein [21, 22]. CREBBP functions as transcriptional coactivators for a large number of DNA-binding transcription factors involved in multiple signaling and developmental pathways. Pasqualucci et al. identified CREBBP/EP300 mutations as a major pathogenetic mechanism shared by common forms of B-NHL [16]. Besides, CREBBP mutations have also been reported to play a role in the initiation and progression of acute lymphoblastic leukemia [15] and diffuse large B-cell lymphoma [17]. All in all, although the three mutations are not sufficient to diagnose PCNSL, vigilance is needed for inflammatory lesions that may eventually develop into PCNSL.

Multiple biopsies and craniotomy were implemented on the patient to confirm the diagnosis because the stereotactic biopsy target site might obscure histopathological diagnosis. Given that the cerebral hematoma was developed on the patient after biopsy and there was no significant remission after symptomatic treatment, lesion resection by craniotomy and hematoma removal were performed. Compared with the results of the first biopsy, the pathological results of excised lesions revealed that CD20 + B-cell infiltration and Ki-67 index were significantly increased. However, it still suggested inflammatory lesion. Due to the formation of a local abscess in the brain tissue, Ki-67 was considered elevated inflammation. The increase in CD20 + B-cell infiltration was insufficient to diagnose PCNSL. Moreover, both biopsy and craniotomy sample showed inflammation lesion. Thus, we ruled out the possibility of stereotactic biopsy obscure histopathological diagnosis.

Four months after total resection of the right frontal lobe lesion, the lesions recurred in the left frontal parietal lobe and were diagnosed as PCNSL. The relatively close time interval between the appearance of the inflammatory lesion in the right frontal lobe and the manifestation of the PCNSL is a characteristic of “sentinel lesions.” Therefore, the question of a false-negative diagnosis of the initial lesion was raised [4]. Because of the cytolytic effect on lymphoma cells of corticosteroids, corticosteroid treatment before craniotomy or biopsy can mask the presence of malignant B-cells and causing inaccurate diagnosis [23]. In our case, although the patient was treated with pulse corticosteroid before craniotomy, two pathological results of the right frontal lobe lesion by different surgical methods (biopsy and craniotomy) all suggested inflammatory lesions. In addition, the genetic test results of the right frontal lobe lesion were insufficient to diagnose PCNSL as well. Thus, the pathological results of right frontal lobe lesions are less likely to be considered false negative.

The initial right frontal lobe lesion had been classified as an inflammatory lesion but of the left frontal parietal-lobe lesion appearing months later which was identified as PCNSL. The histopathological analysis indicated that they truly represent two different pathological entities. It is tempting to speculate on a causal relationship between the inflammatory process and the subsequent PCNSL. From a genetic perspective, the genetic test results of the right frontal lobe lesion showed mutations in RELN, PCLO, and CREBBP genes, which did not diagnose PCNSL. The genetic test results of the left frontal parietal lobe lesion result revealed 23 gene mutations, including MYD88, CD79B, CDKN2A, PIM1, RELN, PCLO, CREBBP, and so on, supporting the diagnosis of diffuse large B-cell lymphoma. As we known, in both genetic tests, mutations in RELN, PCLO, and CREBBP genes were detected, which are related to solid or hematologic tumor development [13–17]. According to three histopathological analyses and two genetic tests, inflammatory lesions were considered the “sentinel lesions” preceding the confirmation of PCNSL.

Many previous studies have reported that heralding inflammatory lesions were pathologically characterized by T-cell dominant lymphocytic infiltration with few B-cell and demyelination [4, 6, 9, 24]. In our case, the first histological examination is consistent with previous studies. Nonetheless, the second histological analysis showed a mixed infiltration of T- and B-lymphocytes. Not only our case but also a few previous case studies have reported a mixture of T- and B-cells without demyelination [4, 25]. Thus, the predominance of T-cell infiltration and demyelinating features are not mandatory characteristics of preceding inflammatory lesions.

The pathogenesis of PCNSL in immunocompetent individuals still remains unclear. It has been hypothesized that PCNSL might emerge unexpectedly due to clonal proliferation among normal B-lymphocytes within the context of an inflammatory CNS disease becoming manifest in “sentinel lesions” [6, 8, 26]. On the other hand, the lymphocytes infiltrating the lesions stated that T-lymphocyte markers, with only rare B-cells, supporting the hypothesis that inflammation affects the resolution of the lesions. Infiltrates of T-cells in B-cell PCNSL lesions have been reported and support a hypothesis that preceding inflammatory lesions could cause difficulty of diagnostic under an inflammatory environment, but it could also be the first immunological response mounted against developing PCNSL. In our case, the first and second histological examinations all revealed the lymphocytes infiltrating the lesions expressed T-lymphocyte markers. In addition, we performed genetic tests on the lesions in two different locations (right frontal lobe and left frontal parietal lobe lesion), and the results showed that the inflammatory lesions (right frontal lobe lesion) had the same three gene mutations as PCNSL (left frontal parietal lobe lesion). Surprisingly, the three mutated genes are associated with solid or hematologic tumor development. It would imply the possibility of PCNSL triggered by preceding inflammatory lesions. These findings of our present study further support the above-mentioned hypothesis.

## Conclusion

In conclusion, when an elder patient has a contrast-enhancing focal lesion, even if the biopsy shows inflammatory changes, the diagnosis of PCNSL should be seriously considered. Moreover, the inflammatory lesions with the same genetic mutation profile as PCNSL have to be valued as lesions with high risk to be PCNSL. Inflammatory brain lesions in old-aged patients have a possibility to be “sentinel lesions” of PCNSL. On pathologic examination, the inflammatory lesions may be a mixture of T- and B-cells, T-cell dominant, and/or demyelinating and distinct from PCNSL. In genetic testing, inflammatory lesions may have the same genetic mutation as PCNSL, which contributes to the early diagnosis of PCNSL. Our present case is the first to demonstrate inflammatory brain lesions heralding PCNSL from genetic and pathological perspectives. It is important not to hesitate to repetitive biopsy for suspicious lesions and to perform genetic testing when conditions permit.
